# Regulator of G protein signaling-1 regulates immune infiltration and macrophage polarization in clear cell renal cell carcinoma

**DOI:** 10.1007/s11255-023-03794-9

**Published:** 2023-09-21

**Authors:** Kun Liu, Dian Xia, Hege Bian, Longfei Peng, Shuxin Dai, Chang Liu, Chao Jiang, Yi Wang, Juan Jin, Liangkuan Bi

**Affiliations:** 1 Department of Urology, The Second Hospital of Anhui Medical University, Hefei, China; 2 School of Basic Medicine, Anhui Medical University, Hefei, China; 3 Department of Urology, Peking University Shenzhen Hospital, Shenzhen, China

**Keywords:** Regulator of G protein signaling-1 (RGS1), Clear cell renal cell carcinoma (ccRCC), Tumor microenvironment (TME), Tumor immunotherapy, M2 macrophages

## Abstract

**Objective:**

To better understand how to clear cell renal cell cancer (ccRCC) is affected by the regulator of G protein signaling-1 (RGS1), its effect on immune infiltration, macrophage polarization, tumor proliferation migration, and to explore whether RGS1 may serve as a marker and therapeutic target for ccRCC.

**Patients and methods:**

In this study, a total of 20 surgical specimens of patients with pathological diagnosis of ccRCC admitted to the Department of Urology of the Second Affiliated Hospital of Anhui Medical University from November 2021 to June 2022 were selected for pathological and protein testing, while the expression of RGS1 in tumors, immune infiltration, and macrophage polarization, particularly M2 macrophage linked to the development of tumor microenvironment (TME), were combined with TGCA database and GO analysis. We also further explored and studied the expression and function of RGS1 in TME, investigated how RGS1 affected tumor growth, migration, apoptosis, and other traits, and initially explored the signaling pathways and mechanisms that RGS1 may affect.

**Results:**

RGS1 was found to be expressed at higher quantities in ccRCC than in normal cells or tissues, according to bioinformatics analysis and preliminary experimental data from this work. Using the TCGA database and GO analysis to describe the expression of RGS1 in a range of tumors, it was found that ccRCC had a much higher level of RGS1 expression than other tumor types. The results of gene enrichment analysis indicated that overexpression of RGS1 may be associated with immune infiltration. The outcomes of in vitro tests revealed that RGS1 overexpression in ccRCC did not significantly alter the proliferation and migration ability of ccRCC, but RGS1 overexpression promoted apoptosis in ccRCC. By in vitro co-culture experiments, RGS1 overexpression inhibited M2 macrophage polarization and also suppressed the Jagged-1/Notch signaling pathway.

**Conclusions:**

RGS1 is highly expressed in ccRCC, while overexpression of RGS1 may increase immune infiltration in the TME and reduce the polarization of M2 macrophages while promoting apoptosis in ccRCC.

**Supplementary Information:**

The online version contains supplementary material available at 10.1007/s11255-023-03794-9.

## Introduction

Renal cell carcinoma (RCC) has been confirmed as the tumor that has been most widely reported in the urinary tract. Existing statistics have suggested that over 400,000 patients of RCC are diagnosed each year worldwide, and there have been 179,368 deaths caused by kidney cancer in 2020 [1], highly jeopardizing patients' lives and health. Over 70% of patients diagnosed with kidney cancer have clear cell RCC (ccRCC) as their post-operative pathology [2]. RCC is insidious in origin and has no specific symptoms at its early stages though early stage RCC is largely clinically curable with surgical treatment. 25% of individuals with restricted RCC experience recurrence or distant metastases following surgery, and 17% of RCC patients are already suffering from distant metastases when they are initially evaluated [3]. Metastatic renal cell carcinoma (mRCC) refers to a form of metastatic RCC [4]. As basic research has been progressing, the treatment of mRCC has been progressively developing in the era of subtractive nephrectomy, cytokine therapy, targeted molecular therapy, as well as immunotherapy [5].

However, the efficacy of cytokine therapy shows a significant correlation with the dose, and the increase of the dose can lead to significantly increased toxic effects of cytokines, such that cytokine therapy is no longer the mainstream clinical treatment [6].

Existing targeted therapies primarily act on the VEGF and mTOR pathways, and they fall into three categories by to their mechanism of action, i.e., tyrosine kinase inhibitors (TKI), mTOR inhibitors, as well as VEGF inhibitors [7, 8].

The major TKI applied currently comprise sunitinib, pegaptanib, axitinib, sorafenib, and cabozantinib, sirolimus, and everolimus serve as the main mTOR inhibitors, and bevacizumab can be the main VEGF inhibitors [7, 9, 10]. Targeted therapy is capable of dramatically extending the overall survival (OS) and progression-free survival (PFS) of patients with mRCC, according to certain clinical evidence. However, targeted therapies have a high rate of adverse effects, and some patients may be required to reduce or stop the drug due to serious adverse effects. Furthermore, it is easy to develop drug resistance after a period of targeted therapies.

Immunotherapy and neoadjuvant immunotherapy have been leaping forward, and PD-1 and CTLA-4 monoclonal antibodies are being employed clinically, and the first-line therapies recommended by mainstream guidelines (e.g., the EAU and AAU) comprise tyrosine kinase inhibitors (TKI) combined with immune checkpoint blockade (ICB), or dual immune checkpoint blockade (anti-PD-1 and anti-CTLA-4) [11]. However, in several clinical trials with PD-1 drugs involved, the objective remission rate (ORR) only reached 20–36%, and the highest complete remission rate (CR) was only 3.6%, especially in mRCC patients, where more than half of the patients administrated with anti-PD-1 did not benefit from the therapy [12]. The majority of mRCC patients do not respond to anti-PD-1 immunotherapy.

Since the proliferation of PD-1 receptors in the TME and the participation of immunosuppressive cells in the TME might cause immunological escape from anti-PD-1 therapy, the TME assumes essential significance [13].

RCC has been reported as one of the most immune-infiltrated solid tumors. As indicated by the results of single-cell sequencing, the microenvironment comprises macrophages, myeloid-derived suppressor cells (MDSC), fibroblasts, CD4+ T cells, CD8+ T cells, and NK cells [14].

Tumor-associated macrophages (TAMs) have been reported as the TME’s essential component and take on great significance to treatment failure, metastasis, as well as the progression of the tumor [15]. There are two primary subtypes of polarized macrophages: M1, classically activated and primarily exhibiting pro-inflammatory and anti-tumor activity; M2, predominantly showing pro-tumor and anti-inflammatory characteristics when alternately activated [16]. TAMs have received a lot of attention recently as potential therapeutic targets for solid tumors, because they are essential regulators of the intricate connections connecting the tumor and the surrounding milieu [17], with significant infiltration of M2 macrophages in tumor patients that show significant correlations with lower survival.

RGS1 refers to a GTPase-activating protein that comprises numerous members [18, 19]. Members of this family are expressed in immune cells [20]. Previous research has indicated that the RGS1 gene is correlated with the chemokine-induced migration of immune cells, thus regulating immune function [21]. Existing research has suggested that RGS1 may be correlated with T cells, whereas the regulation of RGS1 with tumor-associated macrophages has been rarely reported [22]^.^

A wide variety of therapeutic options have been used for advanced RCC. Under this context, insights into the TME should be gained, and drug resistance should be reduced by alleviating side effects. Unfortunately, however, no study has yet explored the role and mechanism of RGS1 in TME of ccRCC. This paper is the first to use integrated bioinformatics and experimental validation to investigate the role of the RGS1 gene in ccRCC, which provides a new perspective on the mechanistic study and clinical treatment of ccRCC.

## Materials and methods

### Raw data

For the gene expression analysis, our researchers evaluated RNA-seq data from 602 ccRCC examples (72 healthy samples and 530 carcinoma samples) as well as directly relevant clinical data from the TCGA database (https://portal.gdc.canceroustumor.gov/). Using R language 3.5.1 and the ESTIMATE algorithm, the proportion of immune-matrix elements in the respective sample TME was assessed using the estimation package, and it was displayed as three scores, i.e., ImmuneScore, StromalScore, and ESTIMATEScore [23]. The above-mentioned scores had a positive correlation with the proportion of immune, matrix, and both, i.e., the proportion rises with the elevation of the respective score.

HK-2 cell line (Pnoxa Bio, China); ACHN cells (Pnoxa Bio, China); 786-O cell line (Pnoxa Bio, China); THP-1 cells (Pnoxa Bio, China); complete MEM medium (KGI Bio, China); complete RPMI-1640 medium (KGI Bio, China); FBS (Gibco, USA). Trypsin–EDTA digest (Solarbio, China); 1 × PBS (0.01 M, pH 7.4) (KGI, China); PMA (MCE, USA); Polyclotamine (Solarbio, China); Lipofectamine 3000 Transfection Reagent (Invitrogen, USA); CCK-8 Assay (KGI Bio, China). Clean bench (BBS-SDC, BIOBASE); medical centrifuge (TD4A, Changsha Yingtai Instruments Co., Ltd.)

### Survival analysis and statistical methods

The software programs Survivorship and survminer were used, along with the R programming language. The log-rank test was used to evaluate whether a difference had reached the role of statistics, and a *p *value of 0.05 indicated that it had. The Kaplan–Meier method was employed to create the survival curves. The clinical markers OS and PFS were employed in this study. In general, oncology OS in clinical practice refers to the OS time of the oncology patient, i.e., the time from the start of the randomization grouping until the death of the patient for any reason. Moreover, the data on oncology OS are usually considered the optimal endpoint for determining efficacy in oncology-associated clinical trials. Whether the sum of the *Z* values of gene expression in malignant tumor tissue was notably elevated in comparison to adjacent normal tissue was examined through the Wilcoxon logarithm test. The Wilcoxon logarithm test was used to determine whether there was a meaningful distinction between neighboring normal tissue and malignant tissue in terms of the sum of gene expression *Z* values. The variability of RGS1 expression at several stages of tumor growth was assessed through the Kruskal–Wallis test. Survival was analyzed (log-rank tests, Cox proportional hazards multiple regressions, and KM graphs). Spearman’s test was performed for related research. All study was conducted using the programming language R (model 3.6.0; R Foundation). A difference that achieved statistical significance was indicated by a two-sided *p* < 0.05.

### Enrichment analysis of KEGG and GO

The tools clusterProfiler, enrich the plot, and ggplot2 were used in R to carry out the enrichment analysis of GO and KEGG. A difference that reached statistical significance was only shown by *p* < 0.05.

### Comparison of test results and clinical phases

The surgical and pathological characteristics of the ccRCC cases were extracted using the TCGA. The study employed R and Wilcoxon rank sum or Kruskal–Wallis rank sum tests, depending on how many medical stages were being compared. SURVIVE, a package for the R language, was loaded for COX regression using single and multiple variables.

### RGS1 and TMB or MSI correlation was looked into.

The R package "maftools" was used to judge somatic cell information (MAF information) from the TCGA human pan-cancer tumor database. For each corresponding malignant tumor, it was calculated how many exon mutations were required to identify TMBs. Using the TCGA database, MSI scores were computed [24]. The interaction between RGS1 expression and TMB or MSI was explored using Spearman mode.

### TIICs and genomic enrichment studies

To comprehend the hyperlink between the expression of RGS1 and immune cell invasion, Spearman correlation analysis was utilized. To estimate TIIC abundance profiles [25], the CIBERSORT computing method was applied for determining all of the tumor samples. Only the NOM *p* < 0.05 genomes showed a difference with statistical significance after GSEA of the full transcriptome for all tumor samples.

### Cell culture and transfection

The HK-2 cell line, ACHN cell line, and 786-O cell line were cultured in MEM complete medium. The cells were subjected to the culturing process in a 5% CO_2_ incubator at 37 °C. Lentiviral packaging carried RGS1 or RGS1-free plasmids. The cells were transfected following the reagent manufacturer's instructions.

### Cell viability

After transfection, cells were divided into normal control, transfected control, and RGS1 overexpressing groups. After the cells had been incubated, 10% CCK-8 was added.

### Western blotting analysis

Prepare the collected cells or tissues and extract the total protein using Western and IP Cell lysis buffer (Beyotime, China) and PMSF configuration. The lysis buffer was introduced into the cells or tissues, and the cells or tissues were completely broken using a cell crusher or tissue grinder. The respective sample protein was subjected to SDS-PAGE gel electrophoresis. Next, the separated proteins were placed on PVDF membranes and closed using skimmed milk, followed by three TBST washes and incubation of secondary antibodies for 1 h at ambient temperature, followed by washing and exposure to the ultrasensitive luminescent solution (Thermo Fisher, USA) for development. The obtained bands were analyzed using ImageJ software. The antibodies used were ACTIN, Caspase3, Cleavedcaspase3, Bcl2, Bax, Jagged-1, and Notch1.

### Flow cytometric analysis

To detect the cell cycle, cells were first molded, then digested with trypsin and collected, the cell suspension was centrifuged for the removal of the supernatant, then washed with pre-chilled PBS and centrifuged to remove the supernatant, and then protected from light. The sample was incubated for 30 min, an assay was performed on the machine, and the data were analyzed. To detect apoptosis, cells were acquired and cleaned using PBS. The cells were resuspended using 500 μl of pre-cooled 1 × Binding Buffer. Annexin V-APC and 10 μl 7-AAD were introduced into the sample. The sample was mixed lightly without light. The assay was performed on a flowmeter.

### Wound-healing assay

Cells were spread evenly in a plate, incubated in an incubator, molded, and continued to be incubated until cells reached 70–80% growth area. Iron-walled cells were administrated with the tip of a 10 μL autoclaved pipette, making an even vertical scratch. Next, the cells were then gently cleaned 3 times using PBS to remove the scratched-down cells. Photographs were recorded at 0 h and 24 h.

### IHC

Tumor and para cancer wax blocks originated from the Pathology Department of the Second Affiliated Hospital of An Medical University, and to repair the antigen, paraffin slices were dewaxed in water and treated with sodium citrate. The sample was incubated in normal goat serum (diluted in PBS) for 10 min at ambient temperature, the serum was decanted, and the sample was not washed. The sample was rinsed with PBS. Next, the sample was incubated at 37 °C and then rinsed with PBS. An appropriate amount of horseradish or alkaline phosphatase-labeled streptavidin working solution was added and then rinsed with PBS. Afterward, the sample developed with DAB chromogen, and it was rinsed well with tap water, re-stained, dehydrated, cleared, and then sealed.

### Real-time PCR

The cells were collected, an appropriate amount of triazole was added, 1/5 chloroform was added, and the tubes were centrifuged at 4 °C at 12,000 rpm. An equal volume of anhydrous ethanol (pre-cooled at 4 °C) was added to the resulting aqueous phase solution and mixed upside down. The precipitate was washed and air-dried; next, it dissolved into enzyme-free water. The RNA concentration was examined and reverse-transcribed. Primer sequences are presented as follows:

β-actin F TGGCACCCAGCACAATGAA.

β-actin R CTAAGTCATAGTCCGCCTAGAAGCA.

RGS1 F CTTGCCAACCAAACTGGTCAA.

RGS1 R TCTCGAGTGCGGAAGTCAAT.

CD163 F GAAATCCCTGCTACTGAACCCC.

CD163 R CAATGGAAACCAGAGAGGAACCC.

### Statistical analysis

All biological experiments were performed at least three times independently. All quantitative data have the expression of mean ± standard deviation. The relevant data were analyzed and then graphed with the use of GraphPad Prism 8.4 software (GraphPad Software, San Diego, CA, USA).

## Results

### Expression of RGS1 in pancytopenia and ccRCC

**Fig. 1:**
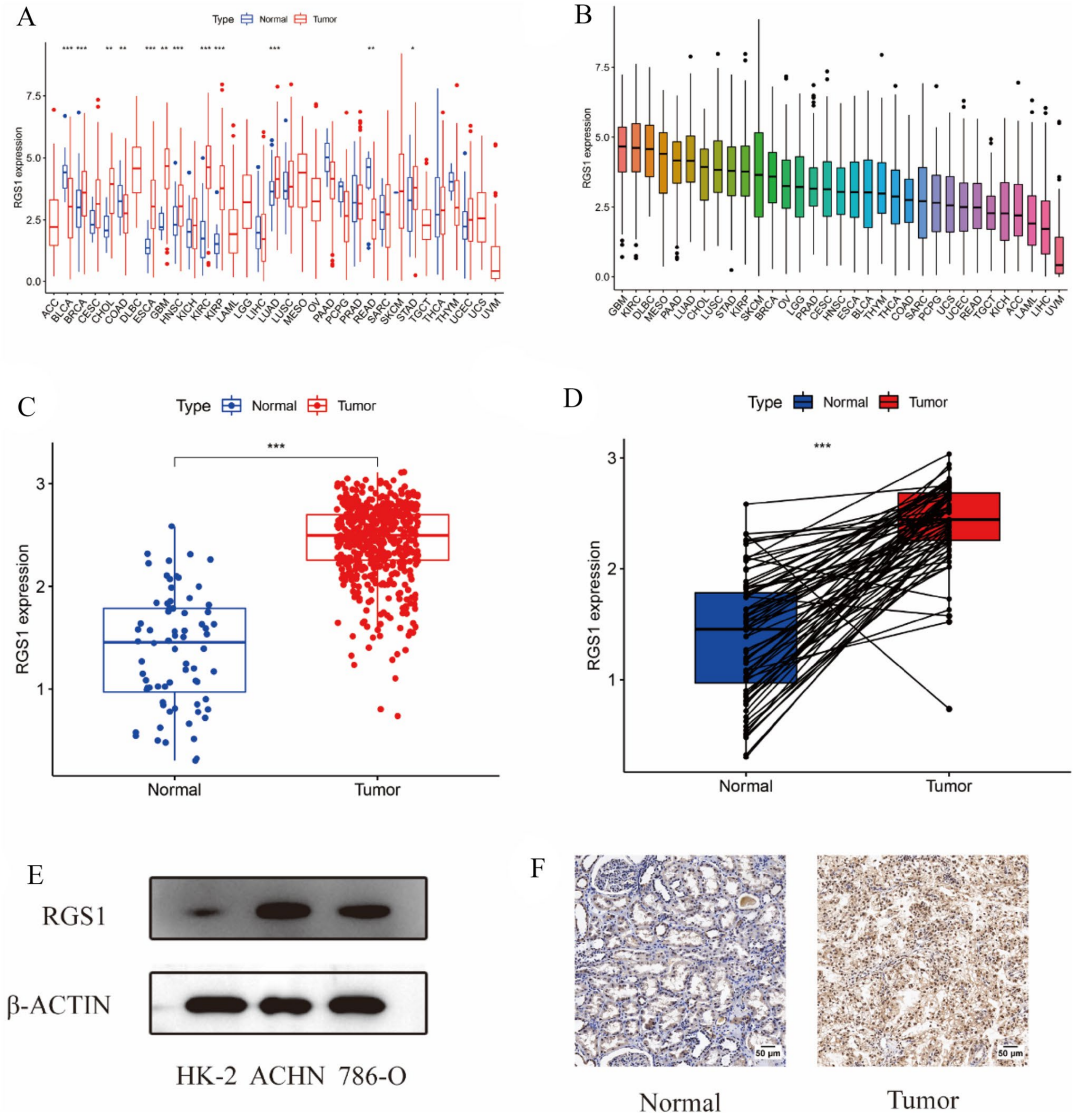
Expression of RGS1 in pancytopenia and ccRCC. **A** mRNA levels in RGS1 pan-cancer in TCGA. Blue represents normal tissues, and red represents malignant tissues. **B** High and low ranking of RGS1 expression in various malignant tumors. **C** RGS1 is expressed differently in healthy tissues compared to those from RCC patients. **D** RGS1 expression in both normal and cancerous organs in a single RCC patient. (E) RGS1 is expressed differently in RCC lines compared to normal renal tubular epithelial cells. **F** Immunohistochemical expression of RGS1 in patients with ccRCC. *p < 0.05; **p < 0.01; ***p < 0.001 compared to the control group

RGS1 expression differences in the TCGA database in a pan-cancer analysis of RGS1 expression differences were first analyzed in this study. RGS1 expression was inconsistent across tumors. Moreover, the normal group of RGS1 in pre-existing tumors and normal tissue pairs were expressed higher than the tumor group in some tumors (e.g., bladder metastatic cell carcinoma, colon cancer, and rectal adenocarcinoma). Besides, several tumors in the normal group had lower RGS1 expression than those in the malignant group. (e.g., breast invasive carcinoma, bile duct carcinoma, oesophageal carcinoma, glioblastoma multiforme, ccRCC, lung adenocarcinoma, and gastric adenocarcinoma) (Fig. 1A). When the gene expression was ranked in order among the tumors, the results showed that glioblastoma multiforme had much greater levels of RGS1 expression than normal tissues, with ccRCC coming in second (Fig. 1B). A significant difference was reported in RGS1 expression in ccRCC. The difference in RGS1 expression in the TCGA was verified using 530 tumor samples and 72 normal samples, and the result indicated that much more RGS1 was expressed in the tumor than within the normal group (Fig. 1C), consistent with the result in the paired analysis (Fig. 1D). WB validation was performed in normal renal tubular epithelial cell line HK-2 cells and two tumor cell lines, ACHN, and 786-O, and the experimental results also suggested that comparing both tumor cell lines to the normal renal tubular epithelial cell line, RGS1 expression was increased to varied degrees in both tumor cell lines (Fig. 1E). Twenty patient samples were selected from the hospital pathology bank, and RGS1 expression in paraffin sections was detected through immunohistochemistry. Furthermore, in all patients observed, compared to the normal group, the tumor group had increased levels of RGS1 expression (Fig. 1F).

### The correlation between RGS1 expression and immunity in ccRCC patients

**Fig. 2:**
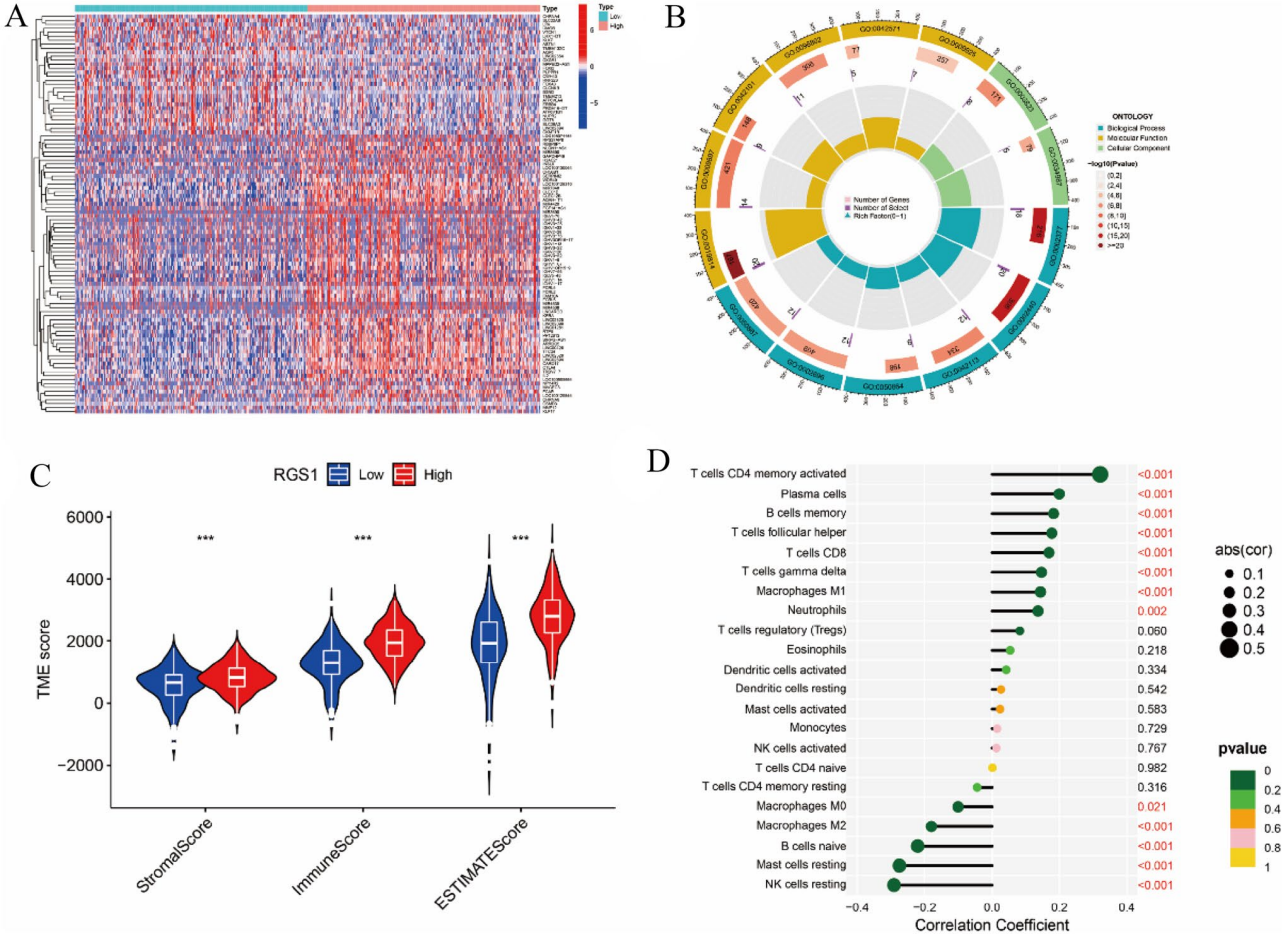
The correlation between RGS1 expression and immunity in ccRCC patients. **A** Heat map representing the genes that are differentially expressed in the TCGA samples between the groups with both elevated and decreased RGS1 expression. **B** Circle plots of GO enrichment studies. **C** Scores for the immune microenvironments of groups with elevated and decreased RGS1 expression. **D** The RGS1 mRNA level and the number of 22 immune cells are correlated, as shown by a forest plot. *p < 0.05; **p < 0.01; ***p < 0.001 compared to the control group

The expression in 530 ccRCC samples was ranked from The Cancer Genome Atlas (TCGA) by high and low expression, in which above median values were defined as high expression, and below median values were defined as low expression. Moreover, the differential analysis was conducted on high- and low-expression patients. The data that achieved statistical differences were retained and made into heat maps (Fig. 2A), and these genes were statistically different in the groups with varying levels of expressiveness, with specific gene names and information shown in Supplementary Table 1. In the GO database, the differentially expressed genes were analyzed in terms of biological processes, molecular function, and cellular composition, and the results are presented (Fig. 2B). The truncation was achieved to score the TME into a stromal cell score, and immune cell score, as well as a combined differential score, which are shown in Supplementary Table 2. The result indicated that differences were identified in different fractions and infiltrations in different groups of high and low RGS1 expression, and the high-expression group tended to have a higher infiltration of highly expressed immune cells (Fig. 2C). A variety of immune cells were subjected to the lollipop plot analysis to analyze the correlation and differential RGS1 expression with cellular components of the immune microenvironment. According to the relevant finding, T cells, B cells, and macrophages were all substantially linked with RGS1 expression. Interestingly, RGS1 expression showed a negative correlation with tumor-associated macrophage M2 (Fig. 2D).

### Prognostic value and enrichment pathway analysis of RGS1

**Fig. 3:**
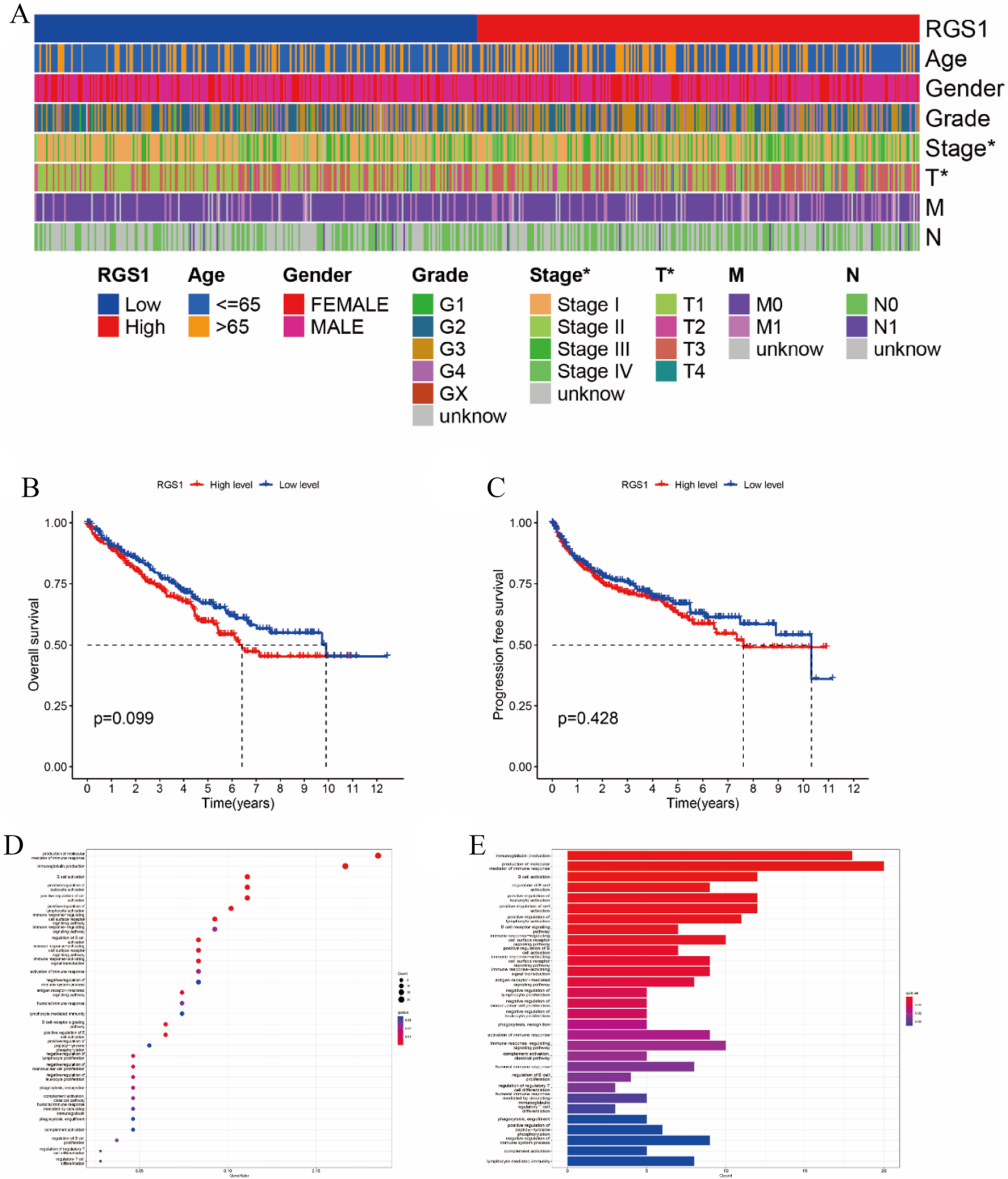
Prognostic value and enrichment pathway analysis of RGS1. **A** Heat map displaying the distribution of clinical traits in the RGS1 groups. **B** Effect of RGS1 with a differential expression on OS in ccRCC patients. **C** Effect of differentially expressed RGS1 on PFS in ccRCC patients. **D** Genes that were differentially expressed in the RGS1 groups were shown using the KEGG pathway analysis of the TCGA database, in a bubble plot. **E** Genes that were differentially expressed in the RGS1 high- and low-expression groups can be seen in the histogram of the TCGA database’s KEGG pathway analysis. *p < 0.05; **p < 0.01; ***p < 0.001 compared to the control group

To ascertain whether RGS1 demonstrates an independent predictive characteristic of ccRCC in this investigation, Cox regression analysis with one and more variables was run. The results suggested that higher RGS1 expression was not correlated with patient age, gender, grade, presence of distant metastases, and lymphatic metastases, and it was correlated with the stage (Fig. 3A). Interestingly, higher expression of RGS1 did not represent higher mortality and the difference between high RGS1 expression and OS did not achieve statistical difference (Fig. 3B). PFS also did not achieve statistical difference RGS1 expression groups, and at the latter stages of the disease, in comparison to the low RGS1 expression group, PFS was greater in the group with high RGS1 expression (Fig. 3C), indicating that RGS1 might have a multifaceted role in ccRCC and potentially act as a protective factor later on. As indicated by the results of the KEGG enrichment study (Fig. 3D, E), DEGs were primarily enriched in the production of immunoglobulin and B-cell activation, two biological mediators of immunological response, and the enriched differentially expressed genes (DEGs) took on critical significance to in TME and immune infiltration in ccRCC, suggesting the important and complex role of RGS1 in TME. As revealed by the above results, RGS1 plays an important and complex role in TME.

### Immune infiltration research between ccRCC subgroups with different RGS1 levels

**Fig. 4:**
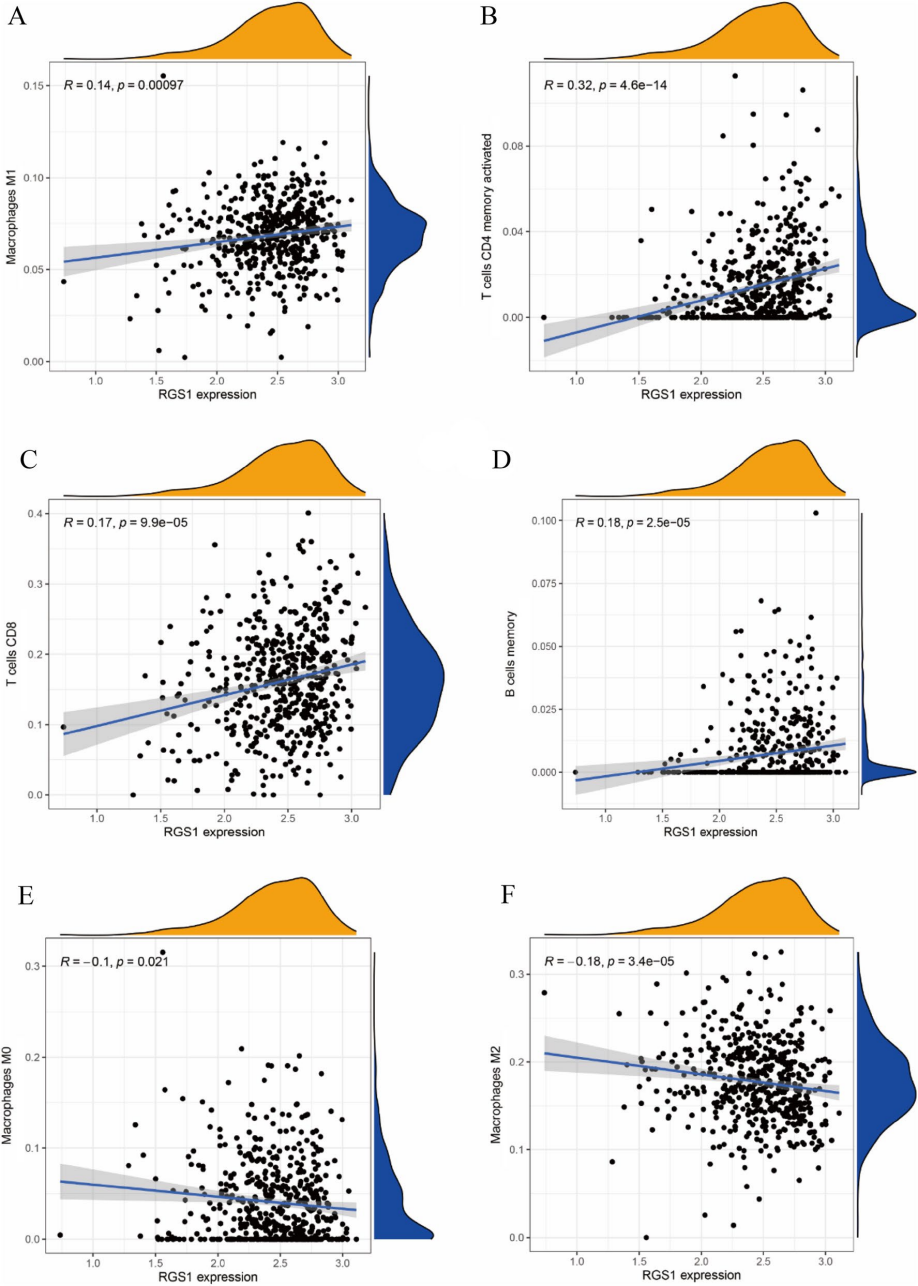
Immune infiltration research between ccRCC subgroups with high and low RGS1 levels. **A**–**E** RGS1 high groups correlate with M1 macrophages, CD4+ T cells, CD8+ T cells, memory B cells, M0 macrophages, and M2 macrophages

RGS1 is critical to immune cells’ activation and differentiation, and the correlation between tumor-infiltrating immune cells (TIICs) and RGS1 overexpression within the ccRCC microenvironment was investigated in depth. The difference in the proportion of RGS1 low and RGS1 high groups among 22 TIICs was analyzed. RGS1 high expression had a positive correlation with M1 macrophages (Fig. 4A), CD4+ T cells (Fig. 4B), CD8+ T cells (Fig. 4C), and memory B cells (Fig. 4D) while showing a negative correlation with M0 macrophages (Fig. 4E) and M2 macrophages (Fig. 4F), with the remaining data are shown in Supplementary Figures.

### RGS1 expression correlates with immune cell infiltration as well as immunotherapy

**Fig. 5:**
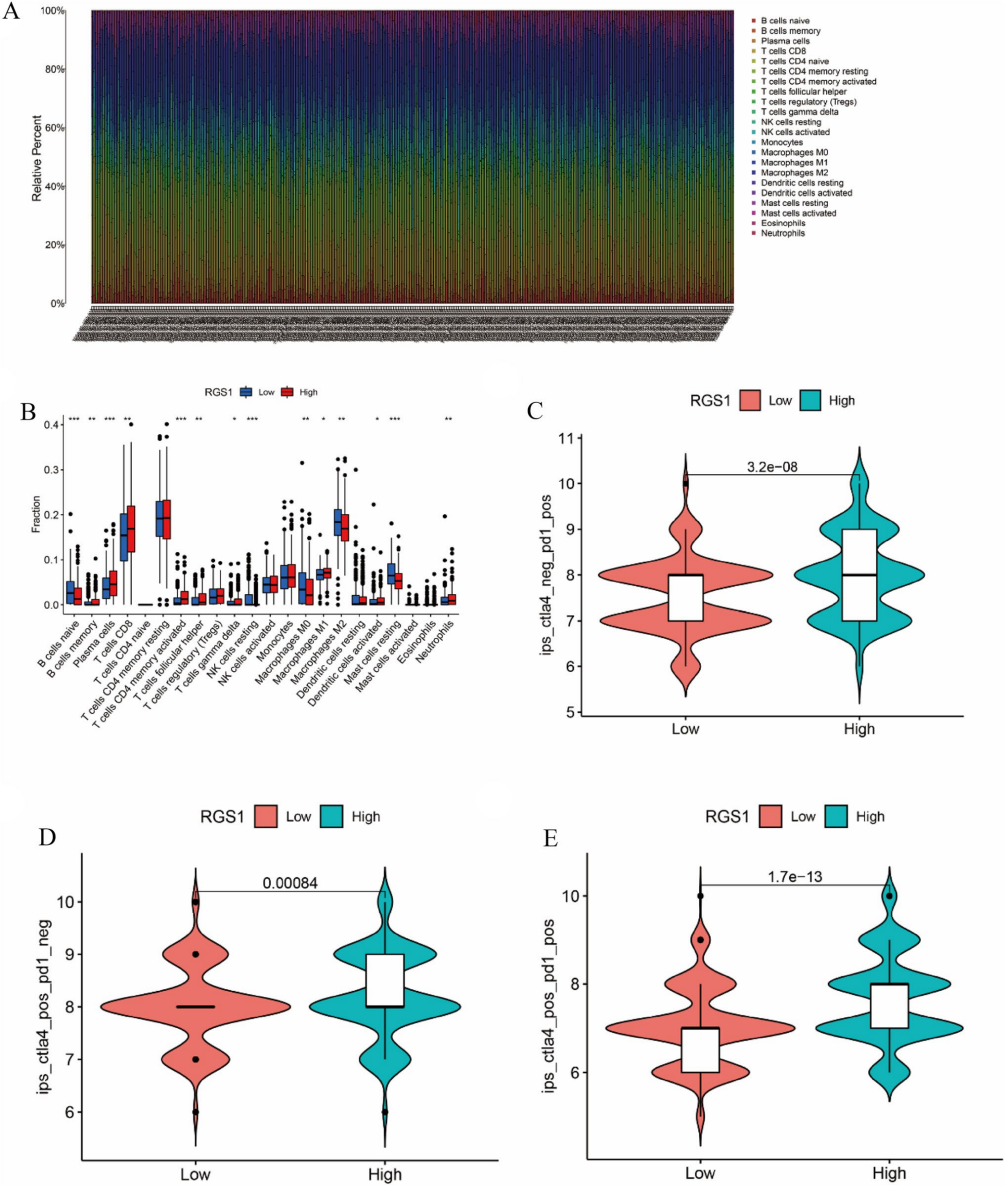
RGS1 expression correlates with immune cell infiltration as well as immunotherapy. **A** Percentage of 22 TIICs in ccRCC samples. **B** TIICs differ in groups with high and low RGS1 expression. **C**–**E** IPS results for the groups with varying levels of RGS1 expression. *p < 0.05; **p < 0.01; ***p < 0.001 compared to the control group

A Pearson relationship test was performed for exploring the association of relevant immune checkpoint genes and RGS1 expression in depth (Fig. 5A, B). According to the study's findings, most immune cells express RGS1, which is connected to this expression. Besides, the Immunoscore (IPS) [26] dataset retrieved from The Cancer Immunome Atlas (TCIA) was used to ascertain if the expression level of RGS1 influences the efficacy of antibody responses against anti-PD-1 and CTLA-4 for a further examination of the connection between RGS1 and immunotherapy reaction. The increased IPS in the group with high RGS1 suggests that the high-expression group had stronger immunotherapeutic effectiveness (Fig. 5C–E).

### Correlation between RGS1 overexpression and proliferation migration of ccRCC

**Fig. 6:**
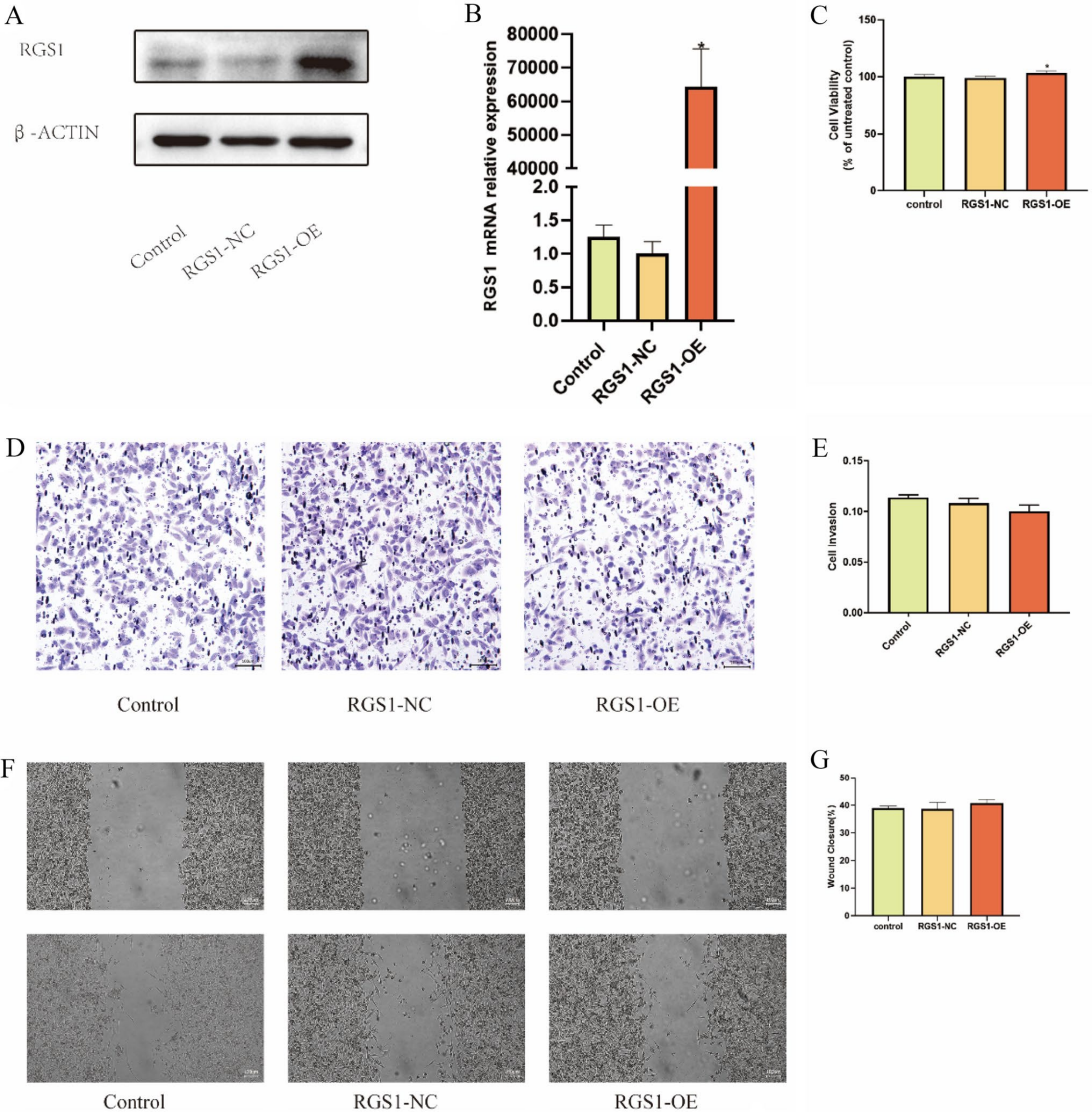
Correlation between RGS1 overexpression and proliferation migration of ccRCC. **A** WB validation of RGS1 overexpression. **B** Real-time PCR validation of RGS1 overexpression. **C** To determine how RGS1 overexpression affects tumor cells and ACHN cell activity, use the CCK-8 test. **D**–**E** To determine the impact of RGS1 overexpression on tumor cell ACHN cell migration and to measure that impact, use the transwell test. **F**–**G** Measurement of the impact of RGS1 overexpression on tumor cell ACHN using a scratch assay. *p < 0.05; **p < 0.01; ***p < 0.001 compared to the control group

RGS1 overexpression lentivirus was set, and the success of lentiviral transfection and overexpression was verified through WB and RT-qPCR (Fig. 6A, B). Next, ACHN cells were assigned to the blank control group, the transfection control group, and the RGS1 overexpression group. As indicated by the result of the CCK-8 assay, the cell viability was improved slightly after RGS1 overexpression, and the difference achieved statistical significance (Fig. 6C). RGS1 overexpression did not lead to the significantly increased or decreased migration ability of RCC cells (Fig. 6D, E). Likewise, the result suggested that RGS1 overexpression did not notably affect cell invasive metastatic ability in the scratch assay (Fig. 6F, G).

### Correlation between RGS1 and ccRCC cycle and apoptosis

**Fig. 7:**
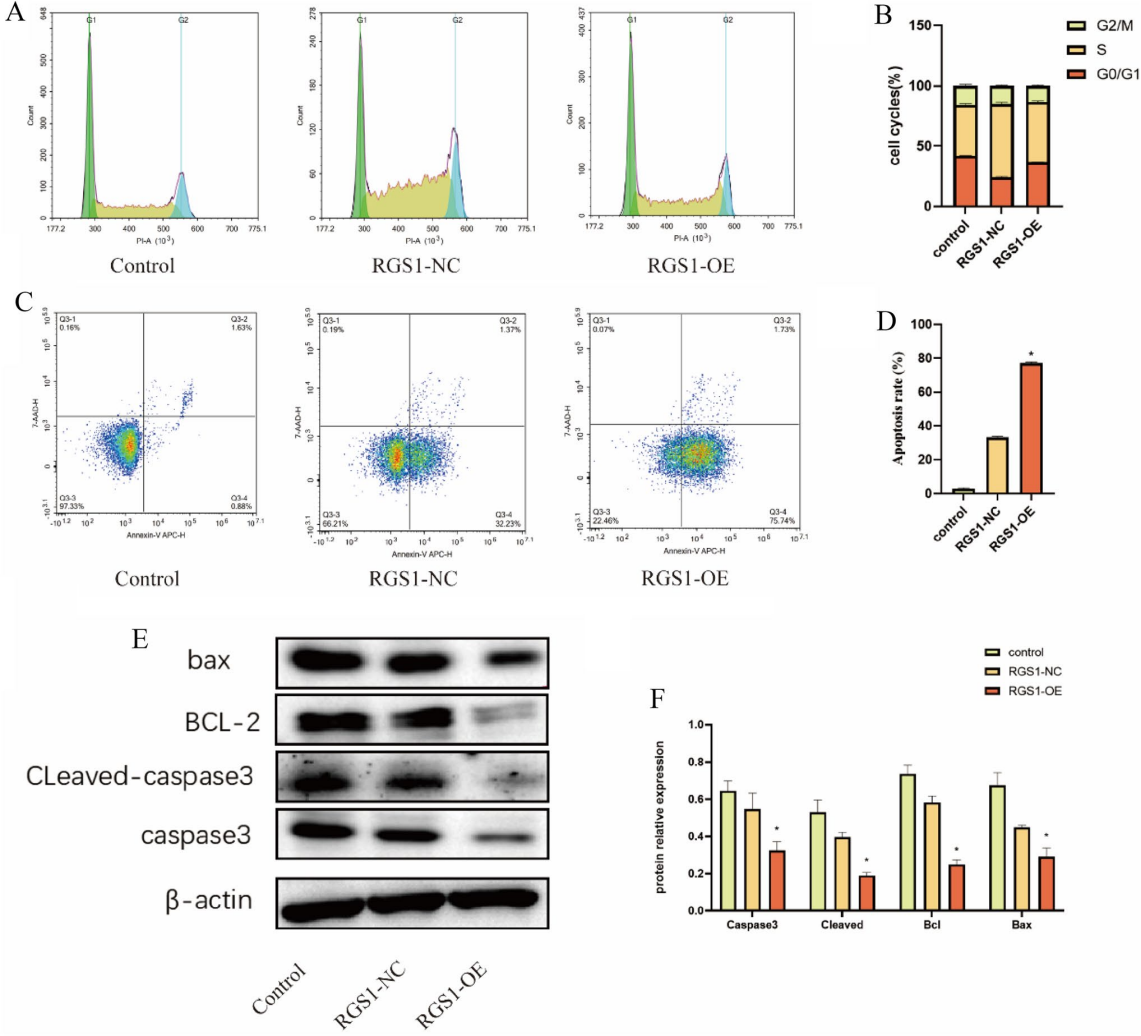
Correlation between RGS1 and ccRCC cycle and apoptosis. **A**–**B** Analysis of the impact of RGS1 overexpression on the cell cycle using flow cytometry. **C**–**D** Detection and measurement of the impact of RGS1 overexpression on apoptosis using flow cytometry. **E**–**F** Effect of RGS1 overexpression on apoptosis-related proteins: WB detection and quantification. *p < 0.05; **p < 0.01; ***p < 0.001 compared to the control group

Through the use of flow cytometry, the impact of RGS1 overexpression on the cell cycle and apoptosis was investigated. As indicated by the experimental results, RGS1 overexpression slightly affected the cycle of ACHN cells (Fig. 7A, B), whereas RGS1 overexpression notably affected apoptosis in ACHN (Fig. 7C, D), and RGS1 overexpression may facilitate apoptosis. Next, apoptosis-associated proteins were detected through WB. The findings revealed that decreased BCL-2 and BAX expression and a decrease in the amount of BCL-2 and BAX dimers caused apoptosis. Interestingly, RGS1 overexpression led to the down-regulated caspase3 expression, which also reduced the expression of the activated form of CLeaved-caspase3. Thus, apoptosis was inhibited, and the overall effect of promoting apoptosis became more pronounced (Fig. 7E, F).

### Effect of RGS1 on macrophage polarization in vitro experiments

**Fig. 8:**
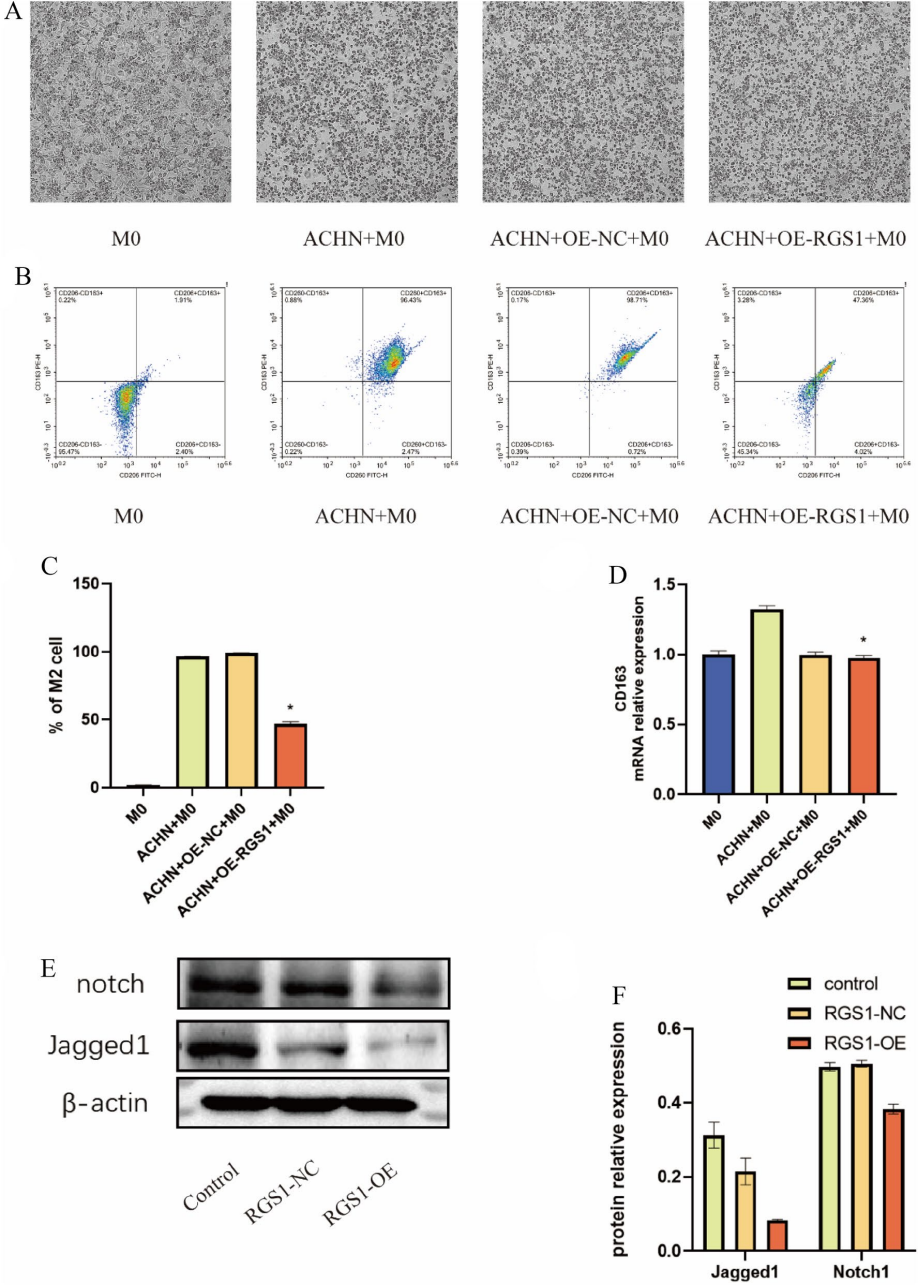
Effect of RGS1 on macrophage polarization in vitro experiments. **A** Effect of co-culture of RGS1 overexpressing ACHN cells and macrophage poles on macrophage polarization. **B**–**C** Flow cytometry detection and quantification of M2 expression after co-culture of ACHN cells and macrophage poles overexpressing RGS1. **D** Real-time PCR assay of co-cultured macrophages polarized to M2 levels. **E**–**F** Effects of RGS1 overexpression on the Notch signaling pathway and quantitative plots. *p < 0.05; **p < 0.01; ***p < 0.001 compared to the control group

Using PMA, THP-1 cells were converted into M0 macrophages, and the cells were assigned to the normal M0 macrophage group, the ACHN + M0 macrophage group, the transfected control ACHN + M0 macrophage group, as well as the RGS1 + M0 macrophage overexpression group (Fig. 8A). Next, the classical markers CD163 and CD206 were detected on the surface of M2 macrophages through flow cytometry, and RGS1 overexpression results in the down-regulated expression in M2 macrophages (Fig. 8B, C). Afterward, RT-qPCR was performed to confirm that RGS1 overexpression similarly led to down-regulated expression in M2 macrophages (Fig. 8D). Previous studies reported that RGS1 overexpression may regulate macrophage polarization through the notch-1/jagged1 signaling pathway [27, 28]. Besides, notch-1 and jagged1 proteins were tested, and the result revealed that RGS1 overexpression may inhibit the notch-1/jagged1 signaling pathway’s expression in ccRCC, exerting a certain effect on tumor-associated macrophage polarization (Fig. 8E, F).

## Discussion

ccRCC refers to a very common urological tumor with a trend of the younger age of onset [29], posing a great psychological and financial burden to patients and their families. Although early stage tumors can be cured by surgery, due to a dearth of early stage screening technologies that are both affordable and easy and specific indicators, many patients are unaware that they should have regular medical examinations., such that some patients were diagnosed in the mid-to-late stages. Currently, targeted therapies and immunotherapy serve as the most effective treatments for advanced RCC [30], but they have many side effects and low response rates, and they still face numerous problems in clinical application [31].

TME refers to an extremely complex and dynamically changing environment in which tumors live [32]. The composition of the TME varies with the type of tumor and largely comprises the environment in which tumor cells live. TME is the site of tumor cell growth and development and then currently recognized by research as a pro-cancer factor. At the early stage of tumor development, tumor cells, and the TME promote and support each other. Early on, the TME gives tumor cells the necessary nutrients for growth and migration while providing an environment that inhibits them from being detected and eliminated by the immune system [33]. Moreover, with the rapid growth of tumors at the later stage, the TME further promotes angiogenesis by secreting growth factors and cytokines to overcome the lack of oxygen and an acidic environment during tumor growth. The TME further facilitates angiogenesis by secreting growth factors and cytokines, overcoming the hypoxic and acidic environment during tumor growth, restoring nutrient and oxygen supply, and removing metabolic waste to improve tumor survival. Accordingly, more research has identified the TME as a novel target for tumor therapy and intervention [34].

The TME is extremely complex, and the role of immune cells in the TME can inhibit tumor formation and exert anti-tumor effects, or promote tumor formation and create an immunosuppressive microenvironment. Immune cells in the TME are activated, chemotactic, and infiltrated after tumor antigen presentation. Neutrophils, macrophages, NK cells, B cells, and T cells all play a huge role in this process [33]. Numerous investigations have demonstrated that TAMs are capable of promoting angiogenesis, suppressing anti-tumor immune responses, boosting the growth of tumors, and secreting various factors regarding extracellular matrix remodeling, such that tumor cell motility and intravascular infiltration can be facilitated. Existing research has suggested that the response of TAMs in the complex TME is largely biased toward the alternative activation phenotype M2, where M2 macrophages up-regulate CD163, interleukin-10 (IL-10), mannose receptors, and arginase-1 (Arg-1) while facilitating tumor neoangiogenesis and suppressing anti-tumor immunity. Besides, M1 macrophages can facilitate tumor neoangiogenesis while suppressing anti-tumor immunity by up-regulating pro-inflammatory molecule expression (iNOS, TNF-α, and IL-1β), thus exhibiting anti-tumor activity. In the TME, prolonged antigen exposure leads to suppression of T-cell activation, reduced proliferation, reduced effector function, and overexpression of multiple inhibitory receptors. As a result, tumor-specific T cells were subjected to significant dysfunction [35], B-cell activation was blocked [36], and M0 macrophages polarized to M2 macrophages, thus leading to tumor progression.

RGS1 is a G protein family member that is mostly expressed in the cytoplasm and cell membrane [37]. Existing research has suggested that the RGS1 gene is correlated with the chemokine-induced migration of immune cells, such that immune function can be regulated [38]. Some research has reported that RGS1 overexpression in breast cancer facilitates breast cancer progression by regulating Treg cells, while RGS1 overexpression in cardiovascular disease affects disease regression by regulating associated macrophages [37]. Siyang Zhang et al. identified the RGS1 gene as a potential target for immunotherapy of cervical cancer [39]. Yunmeng Bai et al. found that the RGS1 gene is a novel marker and promoter of T-cell depletion in a variety of cancers [40]. Di Huang et al. showed that RGS1 plays an important role in tumor immune escape, and targeting RGS1 may provide a new strategy for tumor immunotherapy [41].

The results of this study's preliminary assays and bioinformatics analysis confirmed that RGS1 was expressed more in ccRCC than in healthy cells or tissues. Interestingly, the difference between high RGS1 expression and mortality did not achieve any statistical significance, suggesting that the role of RGS1 in ccRCC is not pure and may even play different roles at different stages of the disease [42]. GO analysis and the TCGA database was used to describe the expression of RGS1 in various tumor types, and the result indicated that RGS1 was significantly expressed in ccRCC. As indicated by the result of the gene enrichment analysis, RGS1 overexpression may be correlated with immune infiltration, and RGS1 can be a marker in ccRCC, whereas RGS1 overexpression can increase immune infiltration of tumors, such that some former cold tumors were converted into hot tumors. Accordingly, RGS1 is promising for the immunotherapy of tumors.

Subsequently, the possible effects of RGS1 overexpression in ccRCC were examined through in vitro experiments. The result suggested that RGS1 overexpression in ccRCC did not significantly alter the proliferative and migratory capacity of ccRCC, and this overexpression had minimum effects on cell activity and cycle. Interestingly, RGS1 overexpression facilitated apoptosis in ccRCC, in contrast to our previous prediction. Moreover, several experiments were performed to ensure the credibility of this finding. The effect of RGS1 overexpression on macrophage polarization and the possible signaling pathways regulating it were further explored through in vitro co-culture experiments.

However, some limitations remained in our experiments. First, the data in this study were primarily acquired from public databases, and it is difficult to validate all immune cells due to the time and conditions of the study. Moreover, a small number of cell lines and clinical samples were selected, which may have led to some bias. Second, the data did not originate from in vivo studies, and it is imperative to conduct animal studies for verifying the reliability of the findings of this study.

## Conclusion

RGS1 is highly expressed in ccRCC, which is related to the staging and grading of ccRCC, and overexpression of RGS1 may increase the immune infiltration of TME and decrease the polarization of M2 macrophages, which may also have important significance for the immunotherapy of ccRCC. Our study provides a new idea for immunotherapy of RCC.

### Supplementary Information

Below is the link to the electronic supplementary material.

Supplementary file1 (XLSX 28 KB)

Supplementary file2 (XLSX 48 KB)

## Data Availability

The [TCGA] repository [https://portal.gdc.cancer.gov/repository] and the [HPA] repository [https://www.proteinatlas.org/] both contain the datasets created and/or analyzed in this study. You can get the remaining information by getting in touch with the writers.
